# GIS-based neural network framework for zoonotic cutaneous leishmaniasis risk mapping in Western Iran

**DOI:** 10.1007/s10661-026-15085-8

**Published:** 2026-03-26

**Authors:** Fatemeh Parto Dezfooli, Mohammad Javad Valadan Zoej, Fahimeh Youssefi, Sudabeh Alatab, Ebrahim Ghaderpour

**Affiliations:** 1 Department of Photogrammetry and Remote Sensing, Faculty of Geodesy and Geomatics Engineering, K.N. Toosi University of Technology, Tehran, 19967-15443 Iran; 2 Institute of Artificial Intelligence, Shaoxing University, 508 West Huancheng Road, Yuecheng District, Shaoxing, 312000 China; 3 Digestive Disease Research Center, Digestive Disease Research Institute, Tehran University of Medical Sciences, Tehran, 1416753955 Iran; 4 Department of Earth Sciences and CERI Research Centre, Sapienza University of Rome, Piazzale Aldo Moro 5, 00185 Rome, Italy

**Keywords:** Vector-borne parasitic disease, Remote sensing, Spatiotemporal modeling, Geospatial Artificial Intelligence (GeoAI)

## Abstract

This study presents a Geospatial Artificial Intelligence (GeoAI) framework for high-resolution Zoonotic Cutaneous Leishmaniasis (ZCL) risk mapping, correlation analysis, and scenario-based projection, integrating geographic information systems (GIS), remote sensing, and neural network architecture. Historical disease maps and multi-temporal satellite-derived environmental layers were jointly modeled using a multilayer perceptron (MLP), two-dimensional convolutional neural networks (2D-CNNs), and three-dimensional CNNs (3D-CNNs). The principal methodological contribution is the implementation of a 3D-CNN, which enables explicit learning of spatiotemporal transmission dynamics. Environmental–disease relationship analyses, based on Pearson coefficients and regression models, identified temperature as the dominant positive environmental driver of ZCL risk. Model performance assessment using root mean square error (RMSE), mean absolute error (MAE), and the area under the receiver operating characteristic curve (AUC) indicates that the 3D-CNN consistently outperforms alternative architectures in capturing complex spatial and temporal patterns. Elevated risk was concentrated in warmer western and southern regions, whereas cooler northern and eastern mountainous areas exhibited lower susceptibility. By 2030, ZCL risk is projected to undergo a spatial shift, with risk decreasing in western regions and intensifying in southern areas, which has direct implications for targeted surveillance and intervention efforts.

## Introduction

Cutaneous leishmaniasis (CL) is a major neglected tropical disease, with an estimated 700,000–1,000,000 new cases reported annually worldwide (Olliaro et al., 2018). The disease occurs in two principal forms: anthroponotic CL (ACL), caused by *Leishmania tropica*, and zoonotic CL (ZCL), caused by *Leishmania major*. ZCL accounts for approximately 80% of reported CL cases globally and represents the dominant transmission form, which is therefore the primary focus of this study (Jahanifard et al., 2020).

ZCL transmission is governed by ecological conditions that facilitate the coexistence of sand fly vectors, human hosts, and rodent reservoirs (Barhoumi et al., 2021; Mohammadbeigi et al., 2021). Numerous studies have reported spatial and temporal heterogeneity in ZCL distribution, with the disease predominantly occurring in hot, low-altitude arid regions and exhibiting peak incidence during summer months (Sharifi et al., 2023; Tabasi & Alesheikh, 2020; Zeb et al., 2021). An earlier study integrated logistic regression with Geographic Information Systems (GIS), demonstrating effective identification of environmental determinants of leishmaniasis in southern Europe (Franco et al., 2011). In Isfahan Province, Iran, statistical and machine-learning (ML) approaches—including decision trees (DT), support vector regression (SVR), and linear regression (LR)—have produced reliable forecasts of CL incidence (Shabanpour et al., 2022). Climate-driven modeling studies further project substantial future increases in CL incidence across North Africa, highlighting the importance of incorporating long-term environmental projections into disease risk assessment frameworks (Saadene & Salhi, 2025). Concurrently, deep-learning architectures integrating convolutional and temporal components, such as convolutional neural networks (CNNs) and long short-term memory (LSTM) networks, have demonstrated superior performance in capturing multitemporal dependencies and advancing spatiotemporal disease-risk modeling (Donizette et al., 2025; Sefrin et al., 2020).

Despite these advances, many epidemiological models still lack full leverage of modern data-driven technologies, even though predictive accuracy strongly depends on high-resolution spatiotemporal inputs. Remote sensing and GIS address this limitation by enabling precise characterization of environmental drivers and supporting geographically targeted intervention planning (Ghaderpour et al., 2025; Youssefi et al., 2022). Geospatial Artificial Intelligence (GeoAI) integrates GIS, remote sensing, and artificial intelligence to analyze and model spatial and spatiotemporal phenomena. Recent advances in this field have substantially improved high-resolution environmental and epidemiological risk assessment (Ahmadi, et al., 2025; Bouguerra et al., 2023; Hasnaoui et al., 2024; Remidi et al., 2025). In this context, Parto Dezfooli et al. (2025) demonstrated that integrating Google Earth Engine–derived remote-sensing data with GIS-based ZCL maps using machine learning frameworks, specifically random forest and extreme gradient boosting, enables accurate disease-risk mapping and identification of priority intervention zones in Ilam Province, Iran. Given that Iran is among the ten countries most affected by ZCL and that Ilam Province exhibits the highest incidence, such targeted risk modeling is essential for evidence-based public health decision-making. Nevertheless, conventional machine-learning approaches remain limited in their ability to capture complex nonlinear spatiotemporal interactions.

To overcome this limitation, the present study advances a GeoAI-based framework that integrates GIS, remote sensing, and neural networks to model complex patterns in high-resolution environmental and epidemiological data. Within this framework, spatiotemporal dynamics are explicitly modeled. Given the limited temporal depth of the available dataset, sequence-based architectures such as LSTM networks are unsuitable; therefore, a 3D-CNN is adopted to efficiently learn spatiotemporal features from short-term image sequences by capturing inter-layer dependencies and temporal context. Overall, the proposed framework characterizes environment–ZCL relationships, improves disease risk modeling accuracy through advanced neural network architectures, enables integrated spatiotemporal analysis via three-dimensional convolution, and provides scenario-based ZCL risk projections for 2030.

## Materials and methods

### Study region

**Fig. 1:**
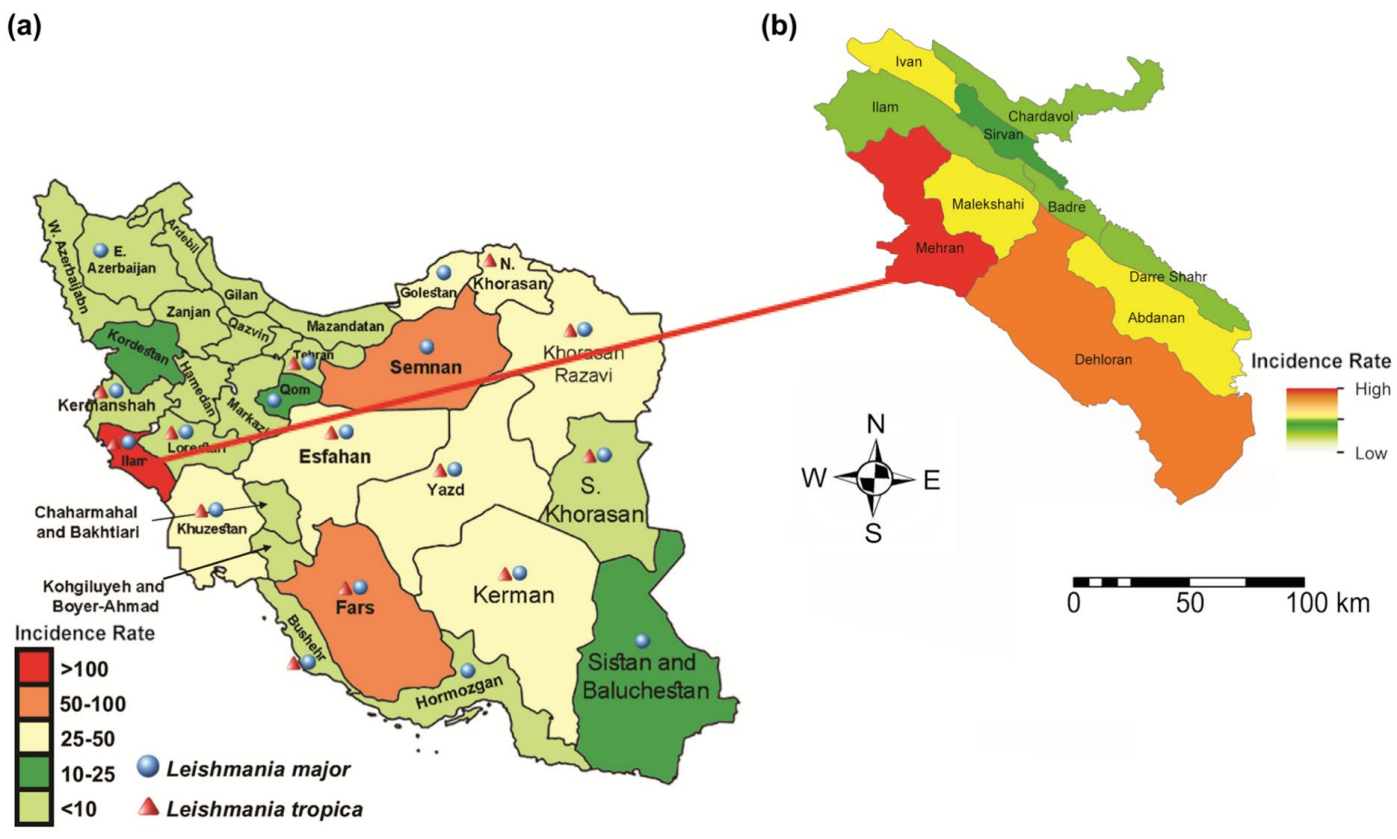
Average incidence of CL: (a) ZCL and ACL in Iran (Sharifi et al., 2023); (b) ZCL in Ilam Province

Ilam Province, located in western Iran, covers approximately 20,133 km^2^ and exhibits pronounced climatic and topographic heterogeneity. The northern and eastern regions are predominantly mountainous, characterized by cooler and semi-humid conditions, whereas the western and southern areas are largely arid, with hot and dry climates (Sharifi et al., 2023). The province’s consistently high ZCL incidence over the past decade, combined with diverse environmental conditions and the availability of six years of georeferenced patient residence data, makes it a particularly suitable setting for spatial epidemiological analysis of ZCL. Figure 1 illustrates the geographic location of the province and the spatial distribution of ZCL incidence (cases per 100,000 population).

### Datasets

The spatial distribution of ZCL was characterized using GIS, while environmental variables were derived from satellite-based remote-sensing products processed on the GEE platform. All datasets were spatially harmonized to a 30 m resolution and temporally aggregated to an annual scale to ensure consistency between response and predictor variables**.**

#### ZCL datasets and mapping

**Fig. 2:**
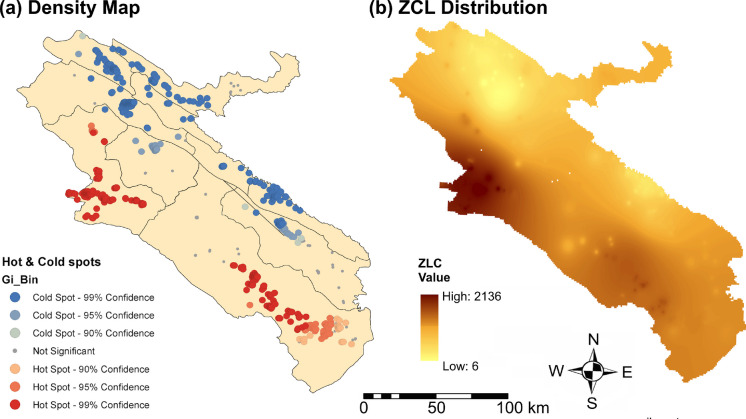
Hot- and cold-spot map (**a**) and density map (**b**) of ZCL distribution in Ilam Province, Iran (2014–2019)

The ZCL dataset comprised 5,353 georeferenced cases recorded between 2014 and 2019, each associated with precise residential coordinates and a documented date of occurrence. Case data were aggregated to an annual temporal scale, yielding six yearly ZCL incidence layers. Initial spatial visualization was conducted using point-based distribution maps and hot- and cold-spot analysis based on the Getis–Ord Gi* statistic to identify statistically significant clustering patterns. A spatially continuous ZCL prevalent surface was subsequently generated using inverse distance weighting (IDW) interpolation in ArcMap, with a power parameter of 2 and 12 nearest neighbors. The interpolated surfaces were produced at a 30 m spatial resolution to ensure consistency with the environmental raster layers. Detailed mapping and preprocessing procedures are described in Parto Dezfooli et al. (2025). For model input preparation, interpolated ZCL values were extracted at the pixel level, such that each raster cell represents the annual ZCL intensity corresponding to the same spatial unit as the environmental predictors. Figure 2 illustrates the spatial distribution of ZCL cases across Ilam Province over the study period (the annual maps are also presented in Appendix B).

#### Environmental factor mapping

**Fig. 3:**
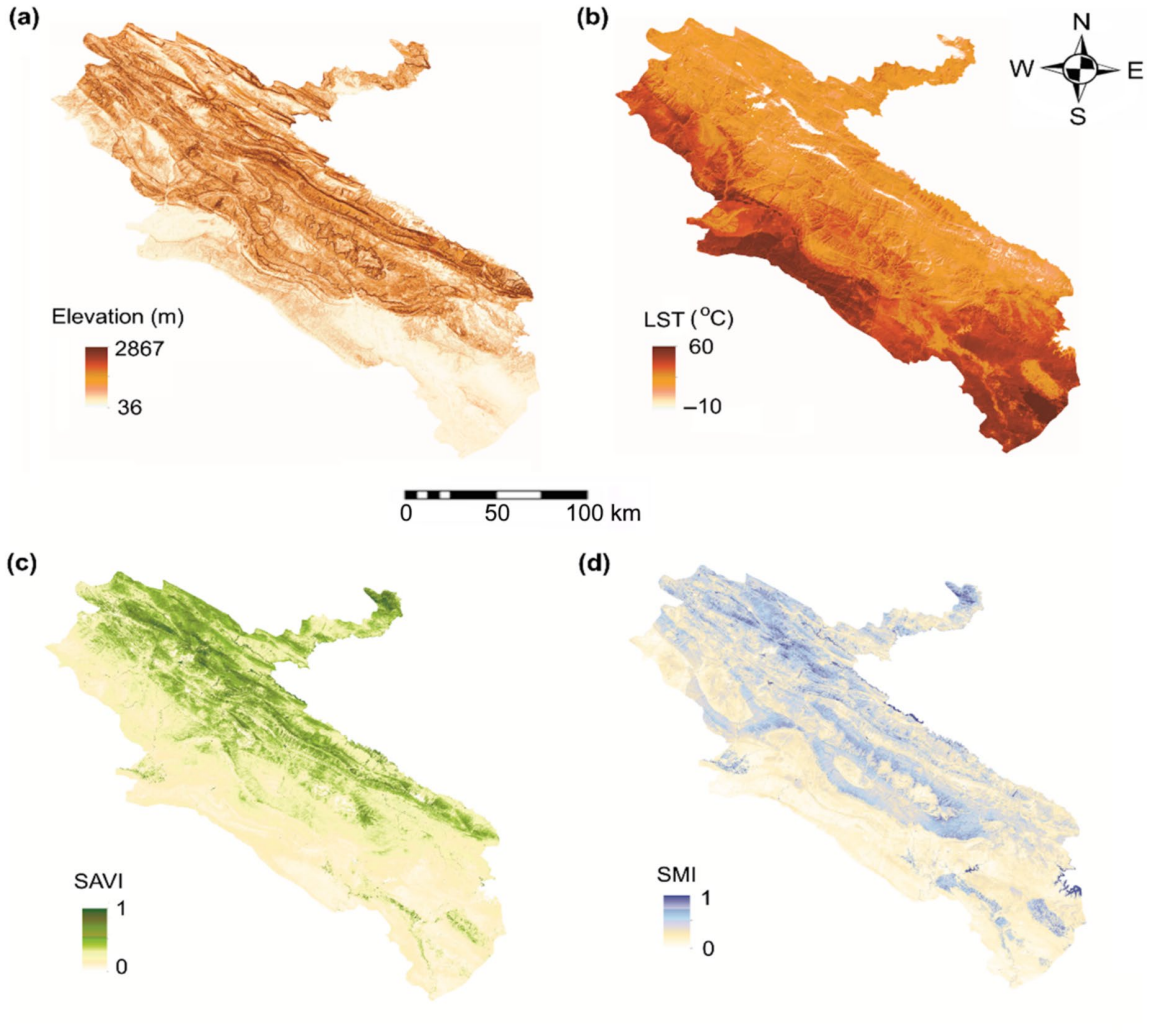
Environmental variables in Ilam Province, Iran (2014–2019): (**a**) DEM; (**b**) LST; (**c**) SAVI; (**d**) SMI

Environmental datasets, constituting the second input group for the neural network models, were derived from satellite-based products processed on the GEE platform for the 2014–2019 period. Dynamic variables, including land surface temperature (LST), soil-adjusted vegetation index (SAVI), and soil moisture index (SMI), were generated from Landsat 8 Level-2 surface reflectance and thermal products at a 30 m spatial resolution and aggregated into annual mean composites following quality control using Landsat quality assurance (QA) bands. Elevation was treated as a static variable and extracted from the Shuttle Radar Topography Mission (SRTM) digital elevation model characterized by a spatial resolution of 1 arc-second (approximately 30 m). The selection of elevation, land cover, temperature, and soil moisture was guided by extensive epidemiological and ecological evidence identifying these variables as key drivers of ZCL risk, as they collectively regulate sand fly habitat suitability, vector activity, and transmission-relevant environmental conditions (AhangarCani & Farnaghi, 2019; Gutiérrez et al., 2024; Kumar et al., 2024; Muñoz Morales et al., 2024). The spatial distributions of all environmental variables are presented in Fig. 3, and complete dataset specifications are provided in Table 1 to ensure transparency and reproducibility.

**Table 1:** Datasets and variables used in the analysis

Variable	Data source	Temporal coverage	Spatial resolution	Unit	Role
ZCL Cases	Health surveillance records (Ilam Province)	Annual (2014–2019)	30 m	Cases per pixel (interpolated)	Response
Elevation	SRTM Digital Elevation Model (DEM)	Static		m	Predictor
LST	Landsat-8 Level-2 Products (GEE)	Annual (2014–2019)		°C	
SAVI				Dimensionless	
SMI					

### Methodology

**Fig. 4:**
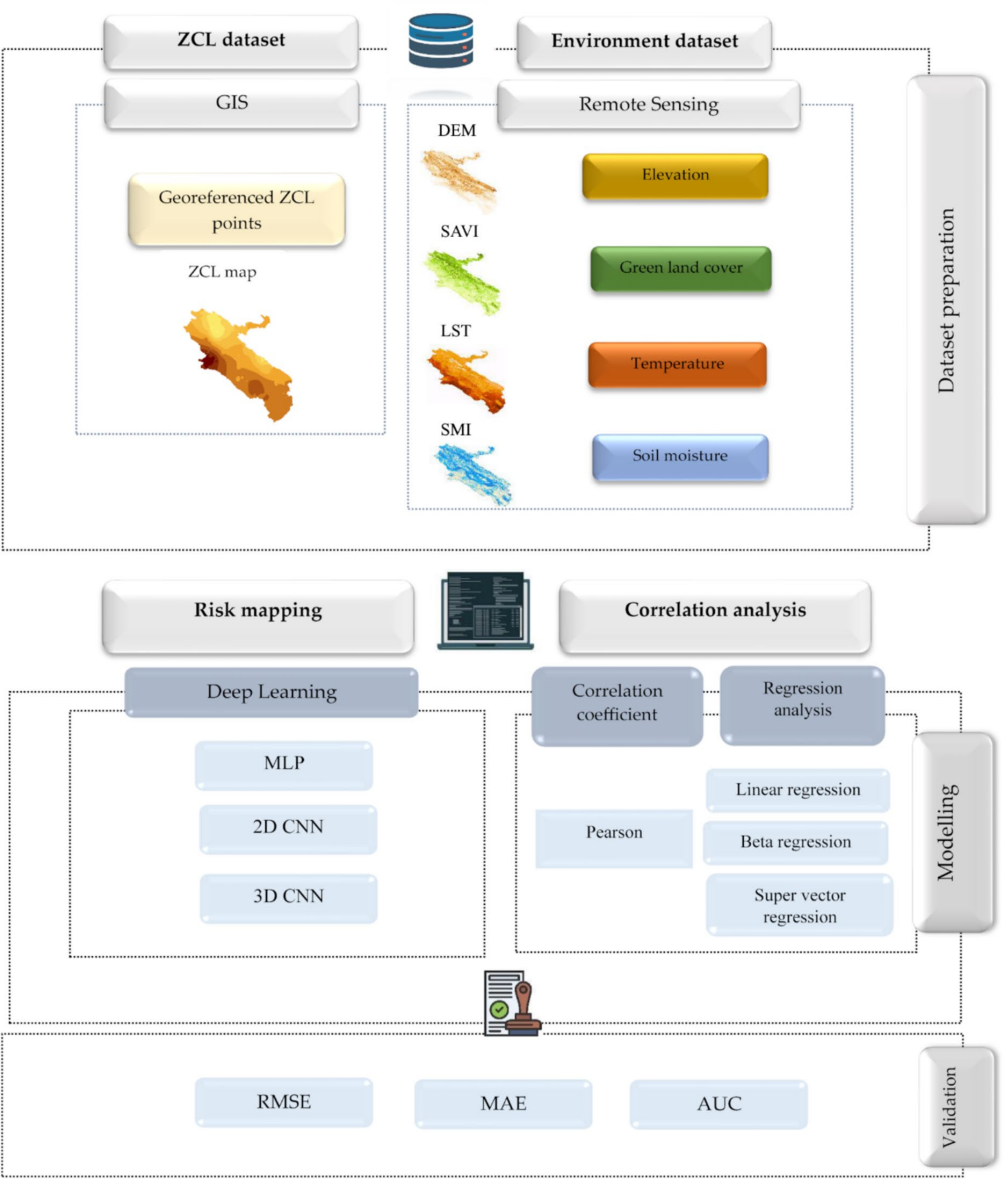
Proposed framework for ZCL risk mapping

This study presents a framework for ZCL risk mapping implemented in three phases: (1) generation of disease and environmental maps as model inputs, (2) statistical analysis of ecological–epidemiological associations, and (3) neural network–driven production of high-resolution predictive risk maps. The overall workflow is summarized in Fig. 4 and described in detail in the following sections.

#### Correlation analysis

For correlation analysis, Pearson’s correlation analysis was initially conducted to quantify the magnitude and direction of linear relationships between environmental variables and ZCL patterns using spatially matched raster–case datasets with ZCL as the response variable (Sedgwick, 2012). Before statistical modeling, all environmental predictors were systematically screened for missing values and standardized using z-score normalization. Subsequently, three complementary regression frameworks were implemented using well-established Python libraries, including NumPy, SciPy, scikit-learn, and stats models, to assess environmental effects under different response formulations. Linear regression (LR) was fitted to standardized predictors with ZCL incidence as the dependent variable to provide a baseline assessment of linear relationships (Su et al., 2012). Beta regression (BR) was applied using normalized ZCL risk values constrained to the (0, 1) interval as the response variable, enabling appropriate modeling of proportional outcomes (Geissinger et al., 2022). Support vector regression (SVR), implemented with a radial basis function kernel, was used to model ZCL incidence as a continuous response, capturing nonlinear relationships while reducing sensitivity to noise through ε-insensitive loss optimization (Liao et al., 2024; Shan, 2023). Model performance was evaluated using the coefficient of determination (R^2^) as a comparative goodness-of-fit metric, reflecting the proportion of variability in ZCL outcomes explained by the environmental predictors.

#### Neural network architectures for ZCL risk assessment

Neural network–based models were adopted due to their capacity to approximate complex nonlinear relations and to hierarchically learn high-dimensional feature representations, making them well-suited for modeling spatial and spatiotemporal dependencies in environmentally driven disease-risk processes. Three complementary neural network architectures were implemented to model distinct dimensions of ZCL risk, encompassing both global nonlinear relationships and explicit spatial and spatiotemporal dependencies.

A multilayer perceptron (MLP) was used as a non-spatial baseline to capture global nonlinear associations among environmental predictors within a fully connected framework. This model provided a reference for evaluating the added value of spatially explicit architectures. ReLU activation and the Adam optimizer ensured stable and efficient training, while dropout and batch normalization improved generalization across heterogeneous environmental conditions (Reyad et al., 2023). Hyperparameters were optimized via grid search, yielding a final architecture with two hidden layers (30 and 15 neurons), a learning rate of 0.01, and 50 training iterations, selected based on minimum mean squared error. All processes were conducted in Python with a fixed random seed (42) to ensure reproducibility.

To explicitly represent spatial heterogeneity, a two-dimensional convolutional neural network (2D-CNN) was implemented using standardized high-resolution GeoTIFF environmental layers (413 × 1013 pixels). Convolutional and max-pooling operations enabled hierarchical extraction of localized spatial features under spatial autocorrelation, while fully connected layers generated a continuous two-dimensional ZCL risk surface, with each pixel representing site-specific incidence or normalized risk (Ouma & Omai, 2023; Remidi et al., 2025). The network architecture consisted of a Conv2D layer with 32 filters and a 3 × 3 kernel, followed by ReLU activation and max pooling. The model was trained using the Adam optimizer (learning rate = 0.01, batch size = 32, 20 epochs) with a fixed random seed (Ogunsanya et al., 2023).

The three-dimensional convolutional neural network (3D-CNN) extended the 2D framework by incorporating the temporal dimension, enabling direct learning of spatiotemporal disease dynamics. Multi-year sequences of environmental predictors and ZCL layers were encoded as volumetric tensors (latitude × longitude × time) and processed through three Conv3D layers with increasing filter depths (32–128) and 3 × 3 × 3 kernels. These filters jointly convolved across spatial neighborhoods and consecutive time steps, allowing the network to learn temporal fluctuations and trends alongside localized spatial patterns. This configuration enabled simultaneous extraction of spatial and temporal features, producing a spatially continuous ZCL risk map that explicitly accounts for spatiotemporal dependencies (Riyanto et al., 2022; Saadat et al., 2022; Varela et al., 2022). Detailed neural network architectures are provided in Appendix A.

#### Validation and comparison

Model performance was evaluated using four complementary statistical metrics: root mean square error (RMSE), mean absolute error (MAE), receiver operating characteristic (ROC) curves, and the area under the ROC curve (AUC) (Chicco et al., 2021; Fang et al., 2024; Yang et al., 2024). RMSE and MAE quantify prediction errors, whereas ROC curves and AUC assess model discriminative ability, together providing a comprehensive evaluation of predictive accuracy and robustness (Bowers & Zhou, 2019).

To evaluate model generalizability and prevent spatial information leakage, spatial k-fold cross-validation (SKCV) was implemented using the GroupKFold strategy in Python. The study area was partitioned into five geographically contiguous and non-overlapping spatial blocks (k = 5) following the approach of Pohjankukka et al. (2017), which served as grouping units within the cross-validation framework. Block boundaries were designed to maximize spatial separation while maintaining an approximately balanced distribution of observations across folds. All ZCL occurrence records and their associated environmental pixels within each block were assigned exclusively to a single fold, with each block used once as the validation set, thereby ensuring strict spatial independence between training and validation data.

**Fig. 5:**
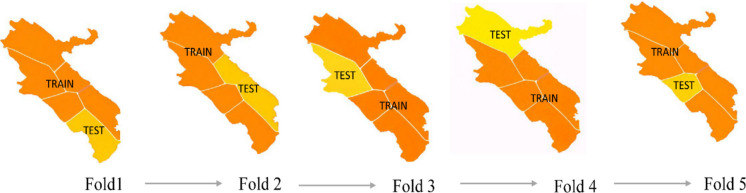
Schematic of the spatial fivefold cross-validation framework

Model performance was evaluated independently for each fold using standard accuracy metrics. Prediction uncertainty was quantified as the pixel-wise standard deviation of model predictions across the five folds, resulting in a spatially explicit uncertainty map. In this map, lower values indicate stable predictions, while higher values highlight regions sensitive to training data variability, typically associated with environmental heterogeneity or data scarcity. A schematic illustration of the SKCV framework is provided in Fig. 5.

#### Risk prediction map for 2030

In this section, a novel, scenario-based disease risk mapping framework for 2030 is presented, integrating high-resolution satellite-derived environmental projections with empirical disease occurrence data. This approach represents a clear departure from conventional risk-mapping paradigms, which predominantly rely on coarse-scale outputs from global and regional climate models (GCMs/RCMs) (Saadene & Salhi, 2025). The framework employs a pixel-wise, linear regression-based forecasting strategy applied to continuous, high-coverage satellite observations spanning 2015–2025 and implemented within the GEE cloud platform. Key environmental indices—including LST, SAVI, and MSI—were projected for 2030. By generating fine-grained, spatially explicit, and temporally consistent environmental projections, this framework enables robust identification of near-future ecological conditions relevant to disease risk dynamics, consistent with the principles demonstrated by Barrett et al. (2020).

Environmental variables exhibit gradual, stable, and directional temporal trends over medium-term periods, according to satellite-based remote sensing studies, rendering them well-suited to quasi-linear modeling (Barrett et al., 2020; Li et al., 2023; Shi et al., 2021). Accordingly, environmental layers for 2030 were generated using robust linear regression applied to annual mean time series. Monotonic trends were estimated using the Theil–Sen estimator, which minimizes sensitivity to anomalous years commonly present in satellite-derived indices and ensures stable extrapolation beyond the observation period (Ghaderpour et al., 2024; Sinsomboonthong et al., 2025). Projection uncertainty was quantified on a per-pixel basis using a non-parametric bootstrap approach (Sinsomboonthong et al., 2025). For each pixel, annual time series were resampled multiple times, Theil–Sen trends were refitted, and corresponding 2030 values were predicted. The resulting distribution of bootstrap-based predictions was used to compute pixel-wise standard deviations, while their spatial mean served as a summary uncertainty metric for each projected environmental layer.

These projected environmental layers were subsequently integrated with the ZCL distribution map using the algorithm identified as most appropriate based on the results of the previous section. The model produces a precise disease risk map that captures complex, non-linear interactions between environmental drivers and disease patterns at high spatiotemporal resolution. Model predictive performance and generalizability were rigorously evaluated using established accuracy metrics and SKCV. Next, pixel-wise standard deviation across spatial folds was calculated to quantify prediction uncertainty, yielding a spatially explicit uncertainty map. This framework presents a scalable and reproducible approach for data-driven risk prediction using integrated regression and deep-learning methods.

## Results

This section presents the results of correlation analysis, risk mapping, and predictive modeling.

### Correlation analysis

**Fig. 6:**
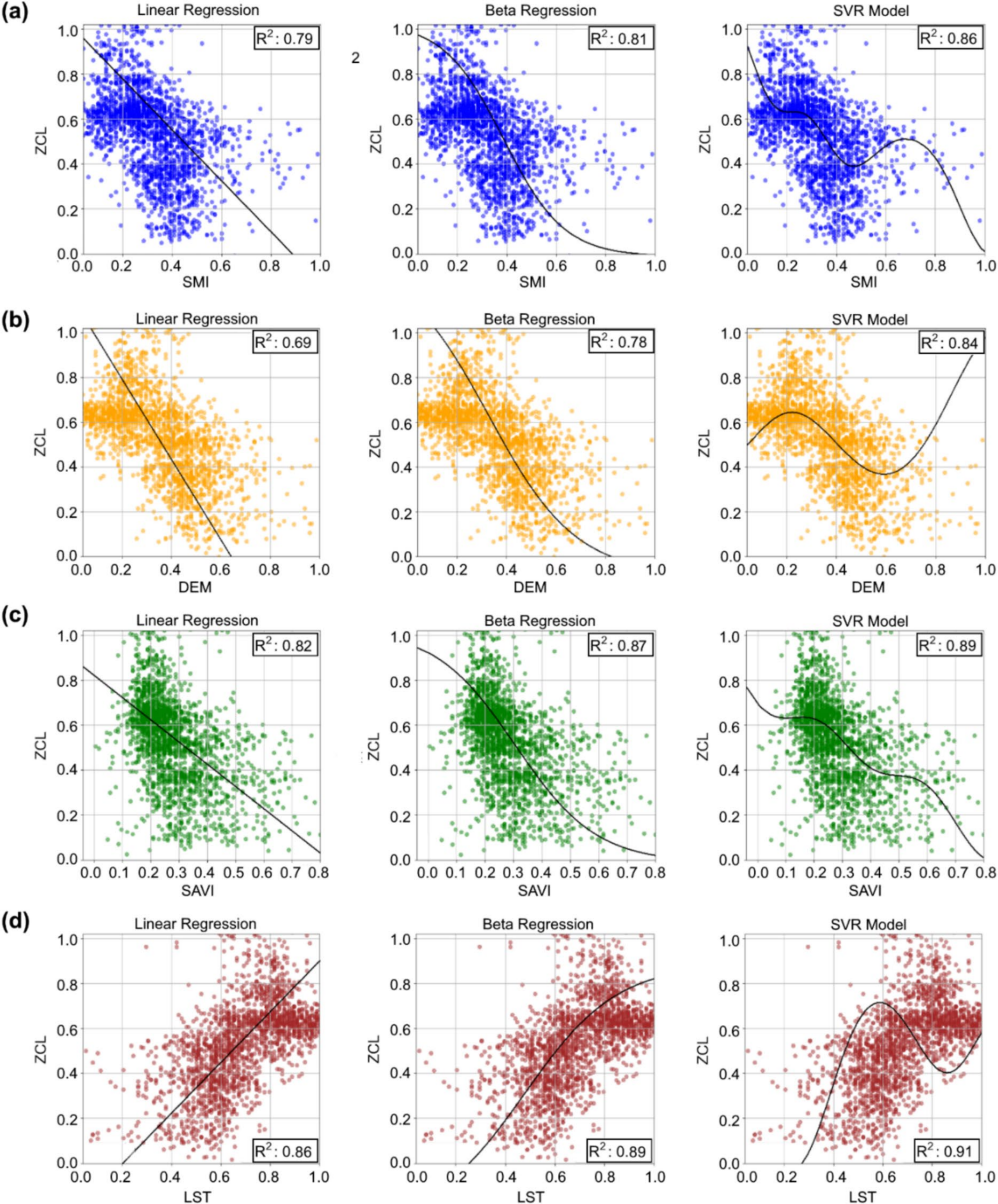
Regression-based correlation analyses between environmental variables and ZCL in Ilam Province, Iran (2014–2019): (**a**) SMI; (**b**) DEM; (**c**) SAVI; (**d**) LST

The relationships between environmental variables and ZCL incidence were examined using Pearson’s correlation analysis and regression analysis. Statistically significant correlations were identified for all variables (p < 0.01). ZCL incidence exhibited negative associations with elevation (r =  − 0.72), green land cover (r =  − 0.81), and soil moisture (r =  − 0.79), and a positive association with temperature (r =  + 0.84). Regression analyses further quantified the variance in ZCL incidence explained by each environmental predictor (Fig. 6a–d). Soil moisture (SMI) yielded R^2^ values of 0.79 (LR), 0.81 (BR), and 0.86 (SVR), while elevation (DEM) achieved 0.69 (LR), 0.78 (BL), and 0.84 (SVR). Green land cover (SAVI) showed similarly high fits (0.82, 0.87, and 0.89), and temperature (LST) provided the best results overall, from 0.86 (LR) and 0.89 (BL) to 0.91 (SVR).

Higher R^2^ values indicate stronger explanatory power of environmental predictors for the spatial variability of ZCL incidence, whereas lower values reflect weaker influence. Across all variables, SVR consistently achieved the highest R^2^ values, followed by BL and LR, underscoring the importance of nonlinear modeling approaches for capturing complex environmental controls on ZCL dynamics. Collectively, the concordant correlation and regression results demonstrate that ZCL occurrence increases under warmer conditions combined with reduced soil moisture, vegetation cover, and elevation, as presented in Table 2 and Fig. 6.

**Table 2:** Pearson correlation coefficients and R^2^ values (2014–2019), Ilam Province, Iran

Environmental factor	*Pearson* $r$	*P value*	${R}^{2}$		
			*LR*	*BL*	*SVR*
Elevation	$-0.72$	< 0.01	0.69	0.78	0.84
Temperature	$0.84$	< 0.01	0.86	0.89	0.91
Green land cover	$-0.81$	< 0.01	0.82	0.87	0.89
Soil moisture	$-0.79$	< 0.01	0.79	0.81	0.86

### Neural network algorithms implementation

**Fig. 7:**
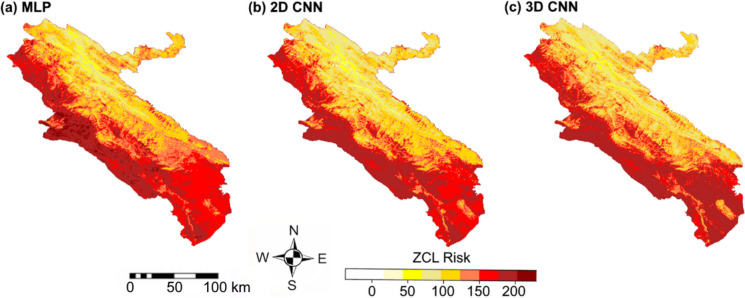
ZCL risk maps produced by (**a**) MLP, (**b**) 2D-CNN, and (**c**) 3D-CNN models in Ilam Province, Iran

Figure 7 indicates that, across all models, high ZCL risk is primarily concentrated in the western and southern desert regions, characterized by high temperatures, low elevations, low soil moisture, and sparse vegetation cover. In contrast, low-risk areas are predominantly located in the northern and eastern mountainous zones. The dominant spatial patterns are largely consistent across the three algorithms. However, the 3D-CNN identifies elevated risk in parts of the southern region and clearly captures interannual shifts in high-risk areas, including a pronounced west-to-south transition of hotspots in 2019, as reported by Parto Dezfooli et al. (2025) (see Appendix B for further details). These results indicate that, by jointly integrating spatial and temporal information, the 3D-CNN more effectively represents the spatiotemporal dynamics of ZCL transmission than conventional modeling approaches.

### Validation and comparison

**Fig. 8:**
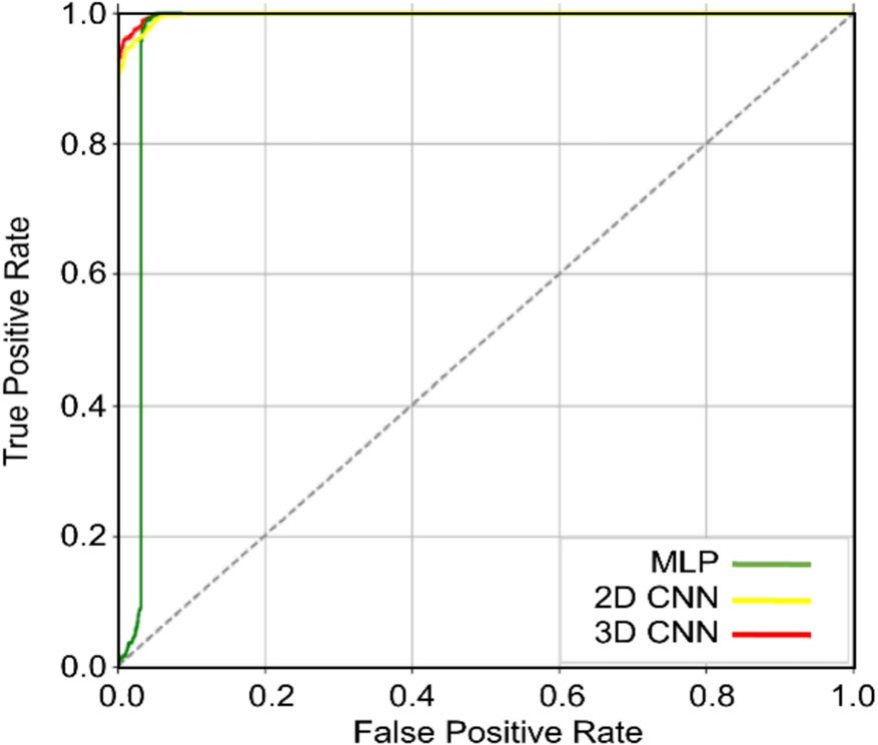
ROC for MLP, 2D CNN, and 3D CNN

Table 3 summarizes the RMSE, MAE, and AUC values for the three algorithms. Among the models, the 3D-CNN achieves the highest overall performance, with the lowest RMSE (14.2304) and MAE (4.0176) and the highest AUC (0.9888), indicating superior predictive accuracy and discriminative ability. The 2D-CNN ranks second (RMSE = 14.2497, MAE = 4.1180, AUC = 0.9884), demonstrating slightly reduced but still robust performance. The MLP exhibits comparatively lower accuracy, with RMSE and MAE values of 14.3125 and 4.1829, respectively, and a notably lower AUC (0.9693), reflecting weaker discriminative capability relative to the CNN-based models. Figure 8 presents the corresponding ROC curves, confirming that models achieve high discriminative accuracy and robust classification performance.

**Table 3:** Comparison of validation metrics (RMSE, MAE, and AUC) across three algorithms

Algorithms	RMSE	MAE	AUC
MLP	14.3125	4.1829	0.9693
2D CNN	14.2497	4.1180	0.9884
3D CNN	14.2304	4.0176	0.9888

The SKCV results demonstrate high consistency in RMSE across all five folds, with inter-fold variability limited to approximately 0.1–0.8 units across models. This stability indicates that the models consistently capture underlying spatial patterns and maintain predictive accuracy when applied to diverse and heterogeneous datasets, underscoring their suitability for robust and reproducible risk modeling in complex ecological and epidemiological contexts.

**Fig. 9:**
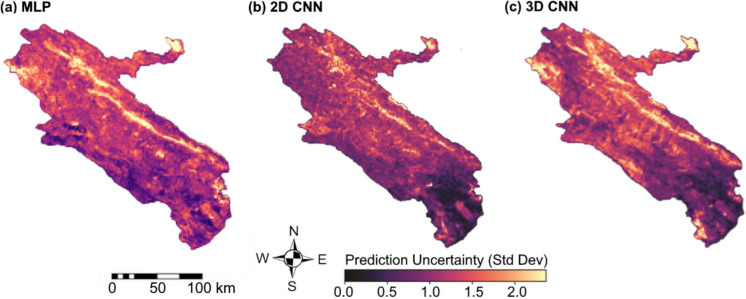
ZCL uncertainty maps by (**a**) MLP, (**b**) 2D-CNN, and (**c**) 3D-CNN models in Ilam Province, Iran

Figure 9 presents uncertainty maps derived from the standard deviation (SD) of model predictions across spatial cross-validation folds. Uncertainty reflects inter-fold variation in model predictions and is highest in areas with sparse ZCL data or pronounced environmental heterogeneity, and lowest in well-sampled high-risk zones. Among the models, the 2D-CNN exhibits the lowest overall uncertainty (mean SD = 0.2176), compared with the MLP (0.3743) and 3D-CNN (0.3056), indicating greater predictive stability. The higher uncertainty observed in the MLP and 3D-CNN reflects their increased sensitivity to data variation. The MLP does not explicitly incorporate spatial context, leading to less stable predictions in data-scarce or environmentally complex regions. In contrast, the 3D-CNN’s greater architectural complexity and temporal integration further amplify its sensitivity to data variation. Overall, the 2D-CNN demonstrates the most reliable predictive performance.

### Risk prediction map for 2030

#### Scenario-based projection of environmental drivers

**Fig. 10:**
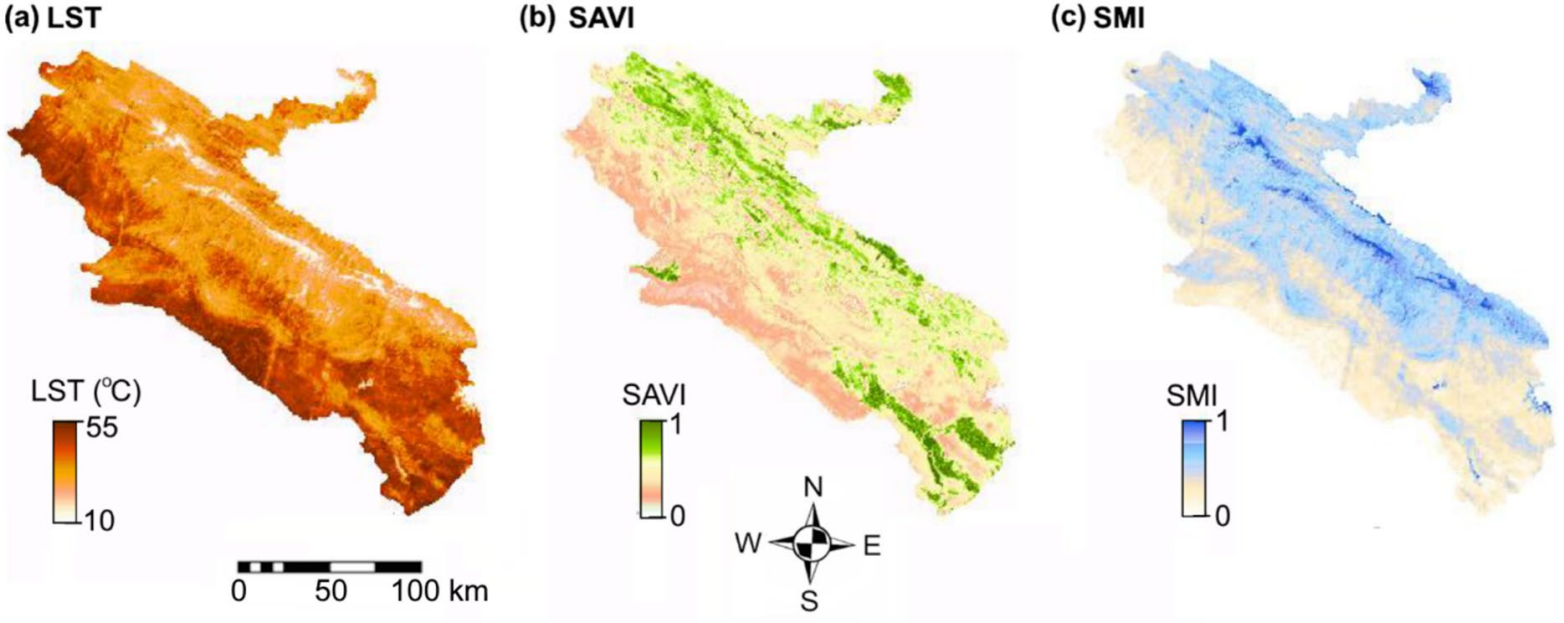
Projected environmental conditions for 2030 in Ilam Province, Iran: (**a**) LST; (**b**) SAVI; (**c**) SMI

Future environmental conditions were generated using scenario-based projections derived from pixel-wise linear trend analysis of annual satellite observations spanning 2015–2025 for LST, SAVI, and SMI. For each pixel, a linear regression model was fitted to the observed time series to estimate the linear temporal trend, which was subsequently extrapolated to infer plausible environmental conditions for 2030. The projected LST, SAVI, and SMI for 2030 are illustrated in Fig. 10a, b, and c, respectively. Projection uncertainty was quantified through bootstrap resampling of the observed time series and summarized as the standard deviation across bootstrap realizations. At the regional scale, uncertainty levels were generally low to moderate, with mean standard deviations of 0.028 for SAVI, 0.62 °C for LST, and 0.031 for SMI. Trend reconstruction accuracy over the historical period was supported by RMSE values of 0.036 (SAVI), 0.78 °C (LST), and 0.039 (SMI). Elevated uncertainty was primarily observed in areas exhibiting high environmental variability or complex spatial gradients. Additional sources of uncertainty include satellite measurement noise, residual atmospheric effects, temporal aggregation of annual means, and the simplifying assumption of linear temporal dynamics.

##### Scenario-based disease risk projection

The disease risk scenario for the year 2030 was generated using a 2D-CNN. The selection of this model for future scenario forecasting was grounded in the results of the preceding analyses and its demonstrated stability and reliability in predictive performance. Application of the algorithm to simulated environmental layers for 2030, together with the available disease distribution maps, yielded mean validation metrics of RMSE = 18.15, MAE = 6.74, and AUC = 0.77. To assess model robustness and to derive the associated uncertainty map, a five-fold SKCV scheme was implemented, revealing an average RMSE variability of approximately 1.6. Collectively, these metrics indicate that the model effectively captures the spatial structure of disease risk while simultaneously reflecting the inherent uncertainty of scenario-based projections.

**Fig. 11:**
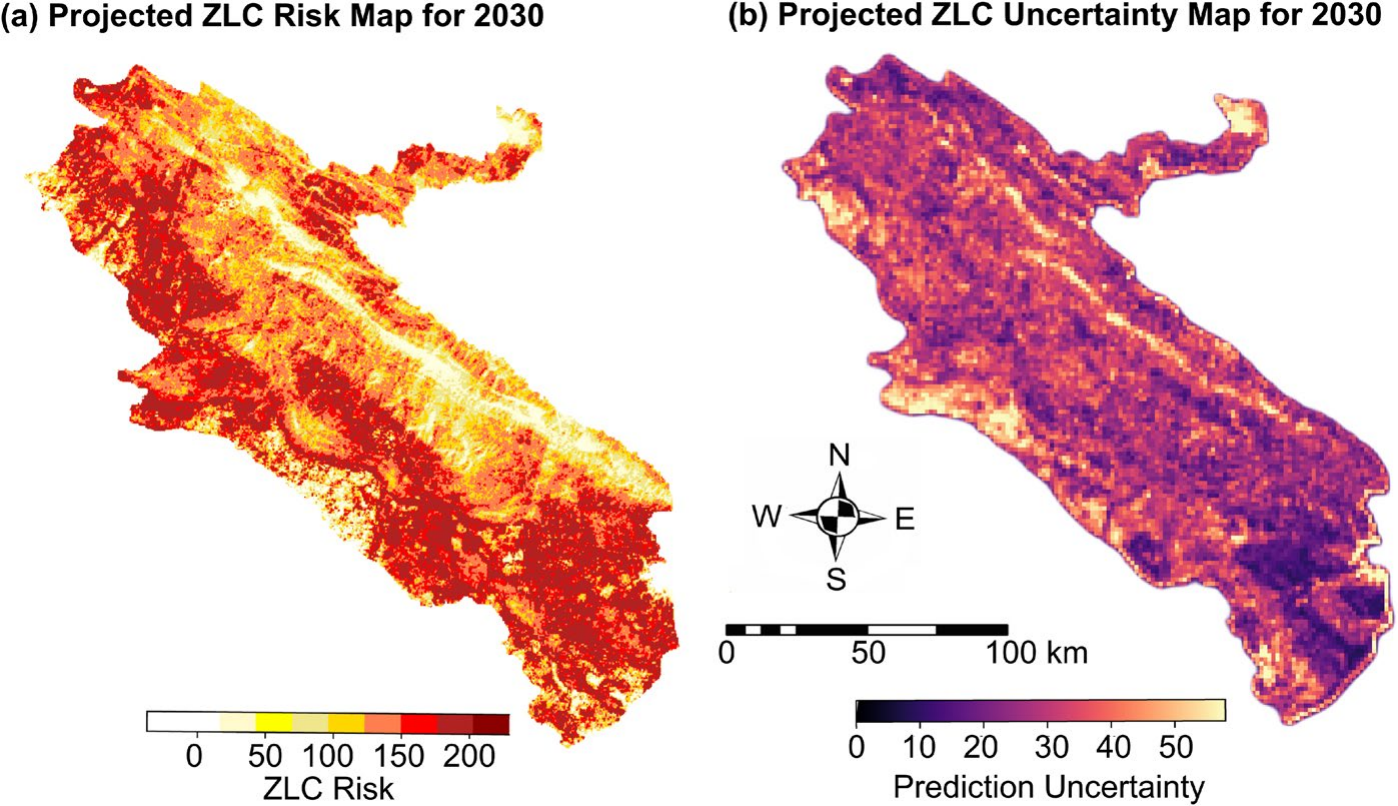
Scenario-based projection of ZCL risk and corresponding spatial uncertainty for 2030 in Ilam Province, Iran

A comparison between the projected 2030 risk map (Fig. 11a) and the risk maps from the preceding analysis (Fig. 8b) indicates a relative intensification of ZCL risk in the southern parts of the province, suggesting a potential spatial redistribution of susceptible areas over the coming decade. The corresponding uncertainty map (Fig. 11b) exhibits a moderate mean predictive standard deviation of 0.4567, with the highest uncertainty concentrated in regions characterized by sparse data availability or pronounced environmental heterogeneity.

Overall, uncertainty levels within high-risk areas remain within acceptable bounds, and the findings demonstrate that integrating a satellite-derived regression approach with the deep-learning framework provides a robust, scalable, and reproducible methodology for scenario-based disease risk assessment.

## Discussion

### Main findings

This study integrates GIS, remote sensing, and artificial neural networks to conduct correlation analysis, high-resolution disease risk mapping, and scenario-based forecasting. The findings reveal strong and statistically significant associations between ZCL incidence and key environmental drivers. Temperature emerges as the dominant positive determinant of disease occurrence, whereas elevation, vegetation cover, and soil moisture consistently exhibit inverse relationships with ZCL incidence. Among the three regression-based models—LR, BR, and SVR—SVR demonstrated the highest explanatory power, highlighting its superior ability to capture complex and nonlinear interactions between environmental variables and disease dynamics. Spatial risk mapping further reveals pronounced clustering of ZCL in low-altitude, arid regions characterized by sparse vegetation cover, whereas cooler, elevated areas exhibit substantially lower risk levels.

In comparison with MLP and two convolutional deep-learning architectures, the 3D-CNN—which explicitly incorporates time as an additional data dimension—demonstrated superior capability in resolving spatiotemporal dependencies and temporal transmission dynamics, thereby achieving the highest predictive accuracy. By contrast, the 2D-CNN produced more stable predictions with comparatively lower uncertainty, particularly within high-risk zones. These results highlight an inherent trade-off between predictive accuracy and model stability, whereby increasing architectural complexity enhances predictive performance while simultaneously increasing sensitivity to environmental heterogeneity and data sparsity. From this perspective, the application of more stable architectures such as the 2D-CNN is methodologically justified and particularly well-suited for scenario-based forecasting.

The scenario-based disease risk map for 2030, generated by integrating satellite-derived linear regression layers with ZCL distribution using a 2D-CNN model, demonstrates a strong capacity to preserve the spatial structure of disease risk while explicitly representing the uncertainty inherent in scenario-driven projections. The predicted risk map indicates a relative intensification of ZCL risk in the southern parts of the province, suggesting a potential spatial redistribution of susceptible areas over the coming decade. The corresponding uncertainty map reveals that elevated uncertainty is concentrated in regions characterized by limited observational data or pronounced environmental heterogeneity. Notably, the western sector of the province, projected to exhibit lower risk levels relative to historical conditions, shows relatively high uncertainty, reflecting underlying environmental variability. These outputs provide robust decision-support tools for the surveillance and management of ZCL in Ilam Province by accurately delineating both persistent and emerging transmission hotspots.

Overall, the proposed GeoAI-driven framework provides a robust, scalable, and reproducible approach for modeling of environmentally driven disease risk. By generating high-resolution risk and uncertainty maps, it serves as an operational tool for evidence-based public health decision-making, enabling authorities to identify priority hotspots, optimize surveillance efforts, and target vector control and preventive interventions in areas of elevated and emerging ZCL risk.

### Comparison with existing literature

The findings are consistent with previous studies that have identified temperature, soil moisture, and vegetation greenness as key environmental determinants of ZCL risk, with temperature emerging as the dominant driver. (Bounoua et al., 2013; Firouraghi et al., 2023; Ramezankhani et al., 2018). The strong performance of the SVR model further corroborates its robustness in modeling complex nonlinear environment–disease relationships (Delbari et al., 2019). In addition, the observed clustering of ZCL risk in low-elevation, arid, and sparsely vegetated areas closely aligns with spatial patterns reported across other endemic regions (Holakouie-Naieni et al., 2017; Mollalo et al., 2015). The neural network results are likewise consistent with uncertainty-aware deep learning theory, whereby increasing model complexity enhances predictive accuracy while amplifying sensitivity to data variability (Basora et al., 2025; Teitelbaum et al., 2024). In line with prior research, CNN-based models demonstrate strong capability for environmental disease risk mapping, while the superior performance of 3D-CNNs highlights their enhanced ability to capture temporal dependencies and complex spatiotemporal epidemiological patterns, outperforming conventional machine-learning approaches (Faraji et al., 2022; McMahon et al., 2021). Taken together, these results align closely with existing literature and further support the suitability of the proposed model.

### Limitations

The proposed methodology provides a scalable and flexible framework for disease risk mapping that is adaptable to diverse geographic contexts; however, several limitations warrant consideration. First, the limited temporal coverage of epidemiological and environmental data constrains the ability to capture long-term trends, lagged climatic effects, and interannual variability in disease dynamics. Second, the analysis was restricted to Ilam Province, which, while enabling detailed local-scale modeling, limits the direct generalizability of the findings to other endemic regions. Additional uncertainty arises from satellite-derived environmental variables and projection models, including sensor resolution, retrieval algorithms, and modeling assumptions, which may propagate into risk estimates—particularly in environmentally heterogeneous or data-sparse areas. Finally, ZCL incidence data were largely obtained from passive surveillance systems, and potential under-reporting or spatial reporting biases may have led to conservative risk estimates in some locations. Collectively, these limitations underscore the need for longer-term datasets, multi-regional analyses, strengthened field-based surveillance, and tighter integration of ground observations with satellite data to enhance future ZCL risk assessments.

### Future research directions

For future research in high-risk areas, establishing a disease information registration system with detailed, geo-referenced patient data would substantially enhance disease surveillance and analysis. Such a system would enable more precise and comprehensive analyses and, when integrated with the proposed modeling framework, facilitate enhanced real-time disease surveillance. Further methodological advances should focus on incorporating additional environmental and socio-demographic variables to improve model robustness and predictive accuracy.

## Conclusions

This study presents an advanced GeoAI framework that integrates remote sensing– and GIS-derived environmental and disease data with neural network models to enable high-resolution ZCL risk mapping. This framework captures both spatial and spatiotemporal disease dynamics, identifies temperature as the dominant environmental driver, and reveals a risk gradient from cooler, vegetated mountainous areas toward hot, arid lowlands, with a potential southward intensification of ZCL risk in Ilam Province by 2030. The results demonstrate the value of spatially explicit ecological monitoring for targeted surveillance and vector control. Nevertheless, the analysis is constrained by the limited temporal coverage of the data and its restriction to a single geographic area. Future research should extend to longer time series and broader geographic domains to further enhance its robustness, transferability, and operational relevance.

## Figures and Tables

**Fig. 12:**
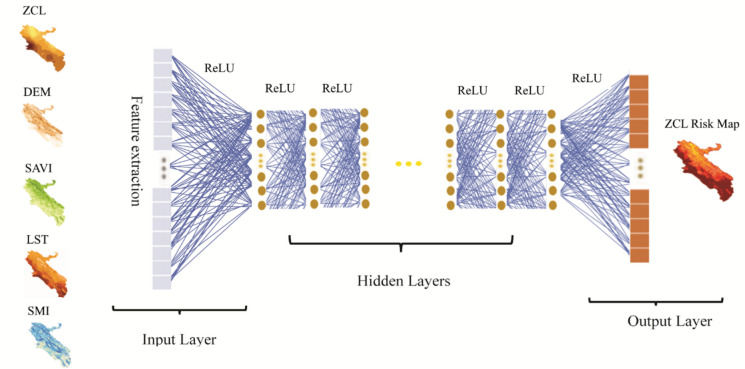
MLP neural network architecture

**Fig. 13:**
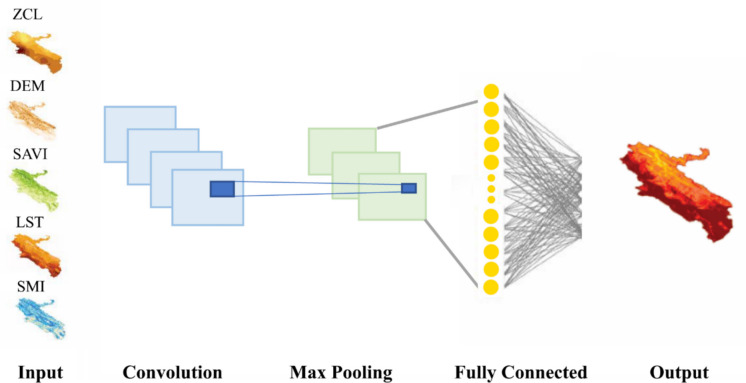
2D-CNN neural network architecture

**Fig. 14:**
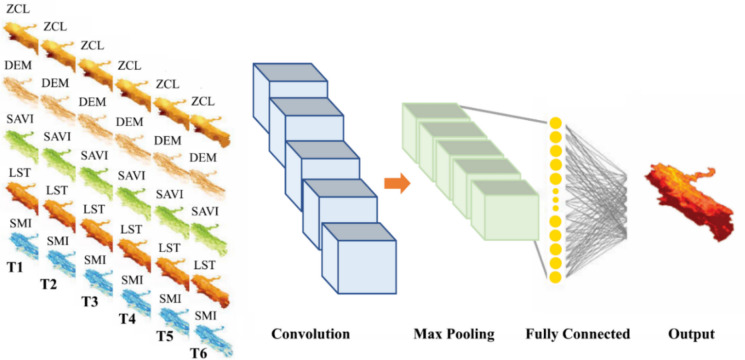
3D-CNN neural network architecture

**Fig. 15:**
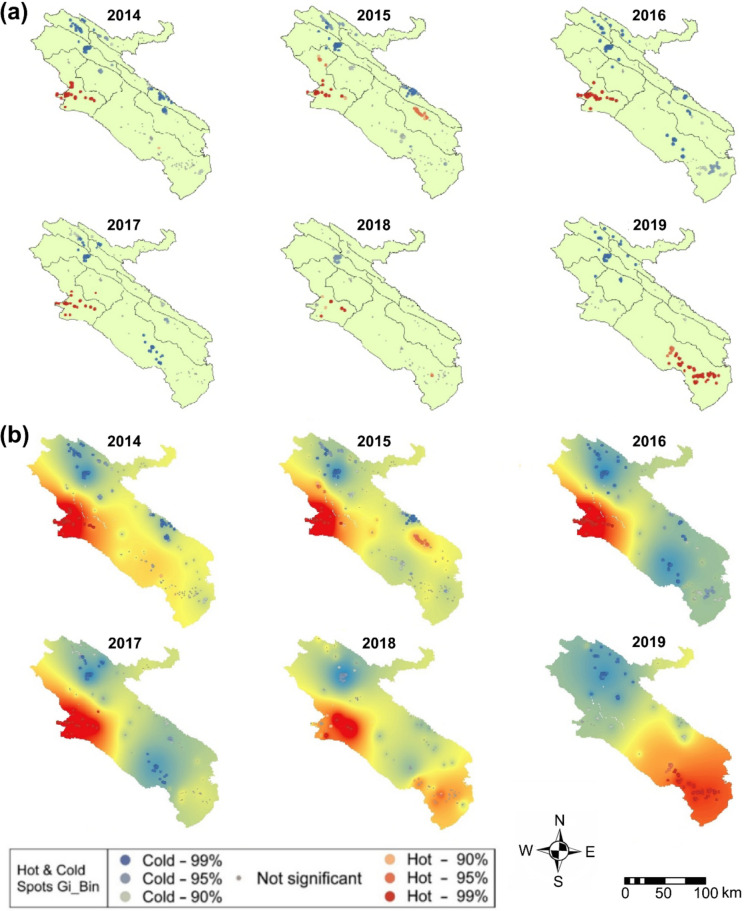
Annual ZCL distribution in Ilam Province, Iran (2014–2019): (**a**) hot/cold spots; (**b**) heat maps

**Fig. 16:**
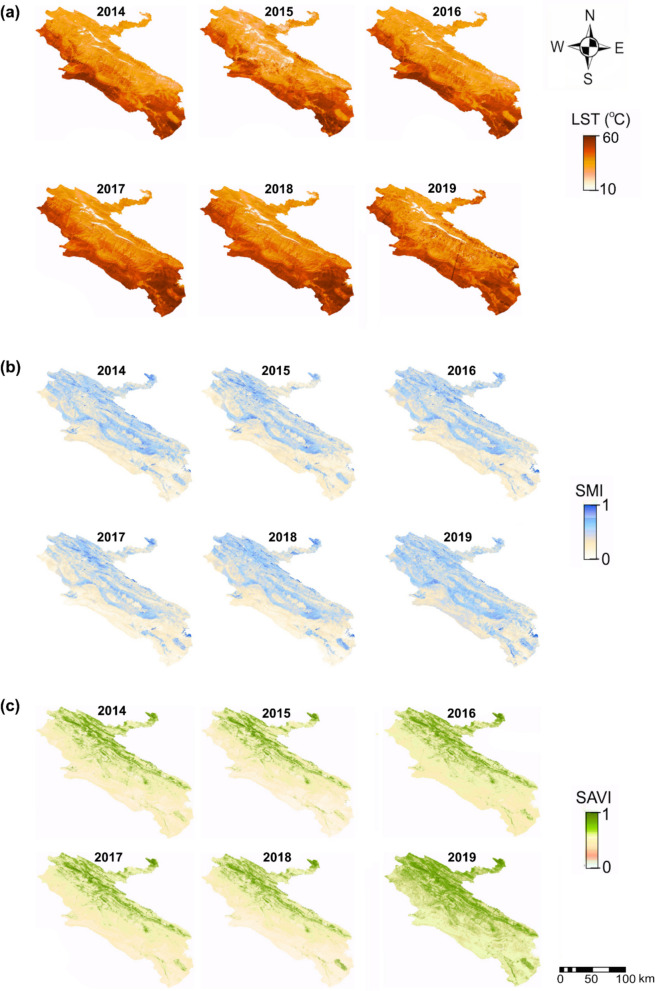
Annual environmental maps of LST, SMI, and SAVI in Ilam Province, Iran (2014–2019)

## Data Availability

Datasets are available at https://github.com/FatemeParto/ZCLRiskMapping

## Appendix A

### Neural network architectures for zoonotic cutaneous leishmaniasis risk assessment

To support reproducibility and methodological transparency, this appendix details the implementation of the neural network architectures used for ZCL risk modeling. Three complementary architectures were employed: a multilayer perceptron (MLP), a two-dimensional convolutional neural network (2D-CNN), and a three-dimensional convolutional neural network (3D-CNN). These models were designed to represent distinct but complementary dimensions of ZCL risk, including global nonlinear associations, localized spatial dependencies, and explicit spatiotemporal dynamics.

Hyperparameter optimization for all architectures was conducted using grid search, systematically evaluating combinations of learning rate, batch size, network depth, and regularization parameters to identify configurations that maximized predictive accuracy and generalizability (Ogunsanya et al., 2023). All experiments were implemented in Python with a fixed random seed (random_state = 42) to ensure reproducibility.

### Multilayer perceptron (MLP)

MLP was implemented as a fully connected feedforward neural network to model global nonlinear relationships between environmental predictors and ZCL incidence. As a non-spatial architecture, the MLP serves as a baseline for evaluating the added predictive value of spatially explicit convolutional models.

Rectified Linear Unit (ReLU) activation functions were applied to all hidden layers, and model optimization was performed using the Adam optimizer to ensure stable convergence. Dropout and batch normalization were incorporated to reduce overfitting and enhance generalization across heterogeneous environmental conditions (Pinkus, 1999; Reyad et al., 2023).

Grid-search optimization yielded a final architecture consisting of two hidden layers with 30 and 15 neurons, respectively, a learning rate of 0.01, and 50 training epochs. The MLP architecture is illustrated in Fig. 12.

### Two-dimensional convolutional neural network (2D-CNN)

To explicitly model spatial heterogeneity in environmental drivers of ZCL, a 2D-CNN was implemented using standardized high-resolution GeoTIFF environmental layers covering the study area (413 × 1013 pixels). This architecture preserves the two-dimensional spatial structure of the input data, enabling direct learning of localized spatial patterns under spatial autocorrelation.

Trainable convolutional filters were used to extract hierarchical spatial features, while max-pooling layers reduced dimensionality and mitigated overfitting. Fully connected layers integrated the extracted features to generate a spatially continuous ZCL risk surface, in which each pixel represents a site-specific incidence or normalized risk estimate (Ouma & Omai, 2023; Remidi et al., 2025).

The final 2D-CNN configuration comprised a Conv2D layer with 32 filters and a 3 × 3 kernel, followed by ReLU activation and max pooling. Model training was performed using the Adam optimizer with a learning rate of 0.01, a batch size of 32, and 20 training epochs. The 2D-CNN architecture is illustrated in Fig. 13.

### Three-dimensional convolutional neural network (3D-CNN)

The 3D-CNN extends the two-dimensional convolutional framework by explicitly incorporating the temporal dimension, enabling direct learning of spatiotemporal disease dynamics from volumetric data. Six-year sequences of environmental predictors and ZCL incidence layers were encoded as three-dimensional tensors (latitude × longitude × time), following the methodology described by Parto Dezfooli et al., (2024, 2025).

The 3D-CNN architecture employed three Conv3D layers with increasing filter depths (32–128) and kernel sizes of 3 × 3 × 3. These kernels jointly convolve across spatial neighborhoods and consecutive time steps, allowing the network to capture localized spatial patterns alongside their temporal evolution. This design supports hierarchical extraction of spatiotemporal features, facilitating robust learning of both short-term fluctuations and interannual trends.

Compared with recurrent architectures, the 3D-CNN provides a computationally efficient and structurally appropriate solution for short temporal sequences, leveraging the continuity of spatial and temporal dimensions without requiring long time series. The model produces a spatially continuous ZCL risk map that explicitly accounts for spatiotemporal dependencies, enhancing predictive accuracy and generalizability in environmental epidemiology applications (Riyanto et al., 2022; Saadat et al., 2022; Varela et al., 2022). The 3D-CNN architecture is illustrated in Fig. 14.

## Appendix B

### Spatiotemporal input maps for the three-dimensional convolutional neural network

This appendix presents the annual zoonotic cutaneous leishmaniasis (ZCL) incidence maps and the corresponding environmental time-series layers used as spatiotemporal inputs to the three-dimensional convolutional neural network (3D-CNN). Together, these datasets enable explicit modeling of spatial heterogeneity and temporal dynamics of ZCL transmission across Ilam Province.

### Annual ZCL maps

Figure 15 illustrates annual maps showing the spatial distribution of reported ZCL cases from 2014 to 2019 across Ilam Province. Several epidemiologically meaningful patterns emerge. ZCL incidence is consistently concentrated in the low-altitude southwestern regions, delineating persistent high-risk transmission zones, whereas substantially lower-case densities are observed in the colder, high-elevation northern and eastern areas.

A pronounced spatial reconfiguration of disease hotspots is evident in 2019, when the dominant focus of ZCL transmission shifted from Mehran—an established hotspot during 2014–2018—to Dehloran, indicating a marked change in outbreak geography**.**

### Annual environmental time-series maps

To characterize environmental drivers of ZCL transmission, annual spatial analyses were conducted using time-series maps of land surface temperature (LST), soil moisture index (SMI), and soil-adjusted vegetation index (SAVI) (Fig. 16). These layers provide spatially explicit representations of the temporal evolution of key ecological factors across the study area.

#### Land surface temperature (LST)

LST maps reveal a pronounced thermal gradient across Ilam Province, with temperatures increasing from the mountainous northern and eastern regions toward the arid lowlands in the south and west. This pattern reflects strong topographic control on surface temperature, with an inverse relationship between elevation and thermal conditions.

#### Soil moisture index (SMI)

SMI maps display an opposing spatial gradient, with soil moisture increasing from arid southwestern zones toward elevated northeastern regions, reflecting a negative association with temperature and a positive association with elevation.

#### Soil-adjusted vegetation index (SAVI)

SAVI maps indicate increasing vegetation density from the hot, moisture-limited southwestern regions to the cooler and more humid northeastern areas. This pattern underscores the critical role of moisture availability in sustaining vegetation productivity and highlights the strong coupling between soil moisture and vegetation dynamics across the landscape.
